# Qualitative insights into the need for a contraception protocol from obstetricians’ and gynecologists’ perspectives

**DOI:** 10.1186/s12905-023-02657-w

**Published:** 2023-09-19

**Authors:** Noha Al Aloola, Shaden Almuneef, Rahaf Alasmari, Huda Al Ewairdhi

**Affiliations:** 1 Department of Clinical Pharmacy, College of Pharmacy, King Saud University, Riyadh, Saudi Arabia; 2 College of Pharmacy, King Saud University, Riyadh, Saudi Arabia

**Keywords:** Contraception, Obstetrics, Gynecologists

## Abstract

**Background:**

Studies on the use of contraception in the Saudi community highlight the need for improving community knowledge about contraception, implementing guidelines, and restricting contraception dispensing. However, there is a lack of studies assessing the need for contraception protocols from obstetricians’ and gynecologists’ (Ob/Gyn) perspectives. This study aimed to assess the need for a contraception protocol from the perspectives of Ob/Gyn physicians.

**Methods:**

Qualitative in depth, semi-structured interviews were conducted with a convenience sample that comprised physicians from the Department of Obstetrics and Gynecology in a tertiary teaching hospital. Interviews were audio recorded and transcribed verbatim and then analyzed using NVivo (QSR International) software.

**Results:**

A total of 12 interviews were conducted and analyzed. Participants indicated a lack of prescribing restrictions and highlighted issues of low contraception literacy in Saudi communities, self-prescribing behaviors, health system organization, and physicians’ knowledge. Participants perceived the need for a contraception protocol guiding the prescribing process and patient counseling without restricting prescribing. Moreover, participants highlighted a number of factors affecting the development and implementation of such a protocol, including the availability of contraception, the need for research by physicians, patient factors, and the expected increased load on the hospital.

**Conclusions:**

This research described current practices, showed the need for a contraception protocol, and highlighted the factors affecting the development and implementation of such a protocol.

## Introduction

Contraception, also known as birth control and fertility control, is the prevention of pregnancy by using various types of methods or devices [1]. Contraception aids in family planning and prevents unintended pregnancies. These methods include nonhormonal and hormonal contraceptive pills, intrauterine devices (IUDs), injectables, implants, female sterilization, male sterilization, condoms, lactation amenorrhea, safe periods, etc. [1].

Women’s use of contraception has been increasing worldwide, including in Saudi Arabia (SA) [2–10]. Saudi Household Health Survey data showed that the prevalence of contraceptive use in SA was 30.4% in 2018 [11]. Women usually take the lead in regulating fertility, and this is due to limited options of contraception methods available for men compared to women [12]. Moreover, some hormonal contraceptives are used for other therapeutic indications, such as in treating acne, hirsutism, menorrhagia, endometriosis, migraine, hyperandrogenism, premenstrual syndrome, polycystic ovarian syndrome, and dysmenorrhea [13–16]. This indeed exposes women to the risk of depression and other adverse effects from contraception. Studies have shown an association between contraception use and depression, anxiety, fatigue, neurotic symptoms, sexual disturbances, compulsive anger, negative menstrual effects, coagulation problems, and even a high risk of breast cancer [17–22].

Contraception use may vary considerably from one society to another. This variation could be related to cultural, educational, or even religious backgrounds. In SA, studies have shown that contraceptive use is influenced by several factors, including maternal age, family size, educational level, and working conditions [10, 23–25]. Recently, SA has shown rapid changes in the sociodemographic features of its population. Women are the most affected by these changes, and with the current Saudi vision of 2030, most women have achieved high education levels and joined the workforce. This has produced changes in attitudes and behaviors regarding contraception [24, 25]. In SA, studies have shown that a high rate of contraception use is associated with a high percentage of side effects. A study conducted in Jazan city in SA reported that the majority of women (87.5%) used oral contraceptives, and most of them (69.6%) had at least one side effect [9]. Another study conducted in Riyadh city in SA revealed that 36% of cerebral venous thrombosis cases among women were secondary to oral contraceptive use [22]. Studies in the Saudi community have highlighted the need for educating the community about contraception, implementing contraception guidelines, restricting oral contraceptives (OCs) dispensed in community pharmacies, and initiating family planning clinics to increase community awareness and proper use of contraception [5–10, 23–26].

According to the World Health Organization (WHO), ensuring access to preferred contraceptive methods for women is essential to supporting the health of mothers and children and a community’s economic situation. The WHO developed two guidelines that help provide the most updated evidence-based family planning services and highlight the importance of implementing these guidelines to improve the quality of care in family planning. These guidelines are medical eligibility criteria (MEC) for contraceptive use and selected practice recommendations (SPRs) for contraceptive use [27, 28]. The MEC provide guidance on the safety of various contraceptive methods for use in the context of specific health conditions and characteristics, whereas SPRs provide guidance on how to use contraceptive methods safely and effectively once they are deemed medically appropriate [27, 28]. Additionally, the WHO published a guide for the integration of these guidelines [29]. This guide presents a structured process to aid countries in incorporating the latest updated MEC and SPR guidance into their national family planning guidelines [29]. However, in SA, there is a lack of studies assessing the current practice of contraception and the need for contraception protocols from physicians’ perspectives. Therefore, this study aimed to assess the need for a contraception protocol in SA from the perspectives of obstetricians’ and gynecologists’ (Ob/Gyn) physicians.

## Materials and methods

Qualitative, semi-structured, in-depth interviews were employed in this study. Physicians from the Department of Obstetrics and Gynecology in a tertiary teaching hospital were invited to participate through email. Invitation letters, participant information statements, and a written consent form were sent to their emails with a request to send a signed consent form as agreement to participate. Interviews were conducted in January 2021 with a convenience sample**.** Each interview took approximately 20–30 min. All interviews were held in English. Interviews were conducted by two members of the research team until data saturation was evident. Data saturation was independently determined by the two members, with a third member of the research team ensuring that the final few interviews yielded no novel information. The construction of the semi-structured interview was based on a literature review [4–10, 23–26]. The interview consisted of four main parts. The first part was demographics, including nationality, years of experience as a physician and Ob/Gyn specialist, number of patients seen per week, and number of Ob/Gyn specialists in their hospital. The second part assessed current practices of contraception prescribing, including contraception prescribing restrictions, procedures performed before prescribing any contraception, information discussed with patients when prescribing contraception, and follow-up processes. The third part assessed the need for a contraception protocol from physicians’ perspectives and included satisfaction with current contraception prescribing practices, difficulties with contraception prescribing, and perceptions of the need for contraception protocols in SA. The last part explored the difficulties in developing and implementing contraception protocols as perceived by physicians. Interviews were conducted in a private setting, depending on the participants’ preferences. The confidentiality of the data was maintained by assigning codes for each participant and their transcript. After participants gave their consent, interviews were audio recorded and transcribed verbatim, after which data were thematically organized and analyzed using NVivo (QSR International) software. The coding of transcripts was conducted by two independent coders, and discussions were conducted to resolve any coding discrepancies and maintain consistency. Triangulation/peer debriefing with an additional researcher was performed to ensure validity.

## Results

**Fig. 1:**
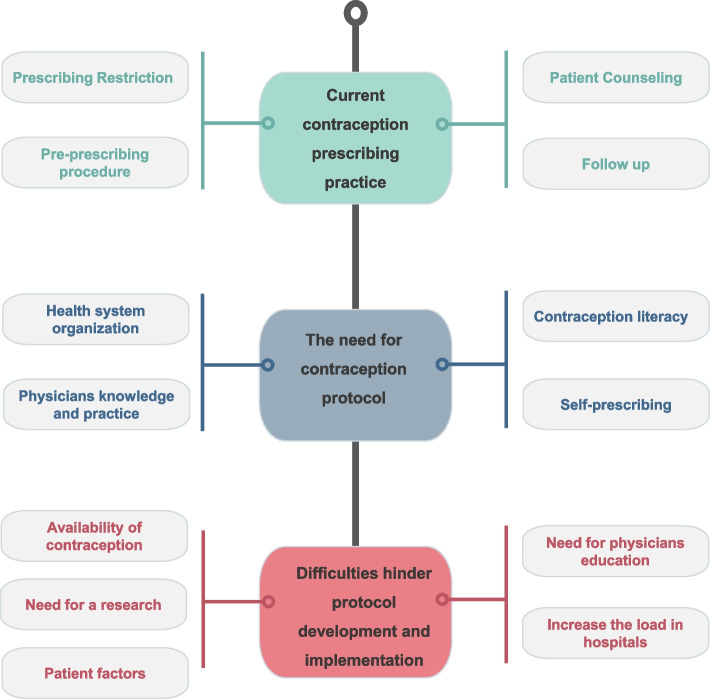
Organizational structure of themes

Twelve interviews were conducted and analyzed. Table 1 shows the demographics of the twelve participants. Thirteen themes pertaining to contraception practices in SA from Ob/Gyn specialists’ perspectives were identified and grouped into three major categories: (1) current contraception prescribing practices, (2) the need for contraception protocols, and (3) difficulties hindering the development and implementation of contraception protocols. Figure 1 depicts the thematic concept map used in this study.

**Table 1:** Participant demographics (*n* = 12)

**Sex**	
Male	6 (50%)
Female	6 (50%)
**Age**	
35 < years old	6 (50%)
36–45 years old	2 (17%)
46–55 years old	1 (8%)
56–65 years old	3 (25%)
**Years of experience as a physician**	
5–9 years	5 (42%)
10–19 years	4 (33%)
> 20 years	3 (25%)
**Years of experience as an Ob/Gyn specialist**	
1–5 years	5 (42%)
6–10 years	3 (25%)
11–19 years	1(8%)
> 20 years	3 (25%)
**Number of patients treated per week**	
< 50 patients	9 (75%)
50–99 patients	3 (25%)
**Nationality**	
Saudi	9 (75%)
Non-Saudi	3 (25%)

### Current contraception prescription practices

#### Theme: prescribing restrictions

The majority of participants highlighted the issue of the lack of restrictions on contraception prescribing. Participants indicated that all family medicine physicians can prescribe all types of contraception. However, some participants indicated that intrauterine devices (IUDs) and implants are restricted to Ob/Gyn specialists.

*“Regarding OCs, any physician can prescribe them; patches and rings are OTC, so anyone can prescribe them. Patients can take implants from the community pharmacy and go to any physician to insert them. Only if the contraception is used for other indications, not for preventing pregnancy, would Ob/Gyn specialists need to prescribe it.” (Participant #2)*

*“No, there is no restriction. Primary care physicians can prescribe only oral contraceptives. IUDs and implants are restricted to Ob/Gyn specialists.” (Participant #6)*

#### Theme: pre-prescription procedure

Almost all the participants indicated that they take full medical histories before they prescribe contraception to rule out any contraindications a patient has. Only some participants indicated that they also perform pregnancy tests to rule out any pregnancy before prescribing, while others just depend on the patient’s history of her periods. None of the participants reported any other hormonal tests routinely ordered before prescribing. Additionally, some participants indicated that ultrasounds were performed only if the participant came for IUD insertion.

“*First, the medical history and physical examination are performed; then we can prescribe to anyone if the patient doesn’t have any contraindication. For OCPs, we do not need tests if we take a medical history. For IUDs or Mirena, some consultants prefer to do ultrasounds or urine pregnancy tests before, but if I know her menstrual period, I can prescribe it without pregnancy tests.” (Participant #2)*

*“A full history is taken, and from the history, it will appear to me if she’s having her period regularly or if she’s on the fifth day of her period; then I don’t need to perform a pregnancy test, and there is no need for hormonal tests. If she’s not known to have high blood pressure, diabetes mellitus, specific rheumatology or some antibodies, then I don’t need to check any of this.” (Participant #10)*

#### Theme: patient counseling

The majority of the participants indicated that they usually counsel their patients about the risks and benefits of contraception. Some participants indicated that they also discuss with their patients the available options, efficacy with failure rate, and the importance of compliance, and some of them ask their patients about their preferences. However, only a few participants indicated that they counsel their patients about drug interactions, and one participant indicated that he counsels his patients about compatibility with breastfeeding.

*“Mainly we discuss the SE, compliance of the patient, and duration of use she wants, which helps to determine the type of contraception. Many patients ask us about the efficacy of each type of contraception.” (Participant #3)*

*“We discuss risks and benefits; additionally, in case we suspect side effects for her, we will be more obvious and mention that to her. You can say it’s verbal consent, which gives information about whatever contraception we’re using, whether it’s hormonal or an IUD. In addition, we tell them about drug interactions, especially for oral contraceptives, as there are certain medications that can interact with these forms of contraception, and they need to take extra precautions.” (Participant #8)*

#### Theme: follow-up

Regarding OC, only some participants indicated that they follow up with their patients after three to six months; other participants see no need to follow up with the patient if the patient is healthy or is familiar with the OC, and they just discuss SEs with their patients and instruct them to come back if they experience any SEs. On the other hand, almost all of the participants indicated that they follow up with their patients one month to six weeks after the insertion of an IUD; then, some follow up with their patients annually, while others do not. Additionally, few participants indicated that they follow up with their patients after progesterone injections every two to three months.

*“It depends on whether a patient uses OCs. We instruct her on how to use them. I don’t follow up with them usually unless they are followed up for another thing. If I am putting in an IUD, I usually call them after one month because complications usually occur in the first month of insertion. If they are fine, then I don’t need to follow up with them.” (Participant #1)*

*“Usually, if she’s on oral contraceptives, there is no need for follow-up. I just give her instructions upon discharge. For an IUD, I follow up with her six weeks after insertion, then there is no need for follow-up unless she has symptoms; she will come back.” (Participant #12)*

### Need for a contraception protocol

Almost all the participants perceived that there was a need for a contraception protocol. However, some participants perceived that this protocol should guide the prescribing process only, without restricting the prescribing, as they perceived that using and choosing the type of contraception method is a right of the patient. Others perceived that this protocol should target the patient counseling process. Furthermore, most of the participants perceived that such protocols should be developed by the Ministry of Health (MOH) to be implemented in the entire country, while some see that as a role of the Saudi Food and Drug Authority (SFDA). Few participants believed that the protocol should be specific for the hospital.

*“I think the protocol should be easy and educational, rather than restrictive, and we have to be careful when we deliver it to avoid affecting women’s right to choose and access.” (Participant #1)*

*“I think it is important but not for restricting prescribing. I am against restricting prescribing. I believe that any woman has the right to get contraception when she needs it without any restrictions, but she should know about the side effects, prerequisites, and contraindications beforehand. We need a protocol for the prescribing process when she goes to the pharmacy; the pharmacist should ask her about contraindications and educate her about the use and side effects.” (Participant #2)*

*“There is a lack of protocol, not in the references but in the clinic itself. It is better to have a table in the clinic where you can explain to the patient and then choose according to the table . This can guide you and make you certain.” (Participant #9)*

*“I think contraception should be easily accessible, with special restrictions if the patient has contraindications. Many patients take contraception without proper assessment. I think we need a proper protocol and some kind of restriction regarding prescribing some types of contraception because some patients have liver disease and they take OCs for a long time.” (Participant #3)*

Participants relayed the need regarding different issues related to patients, health systems, and physicians. These included low community literacy on contraception, self-prescribing, health system organization, and physicians’ knowledge and practices regarding contraception.

#### Theme: contraception literacy

Most of the participants highlighted the issue of low knowledge among most patients regarding different types of contraception and the effects of communities’, families’, and relatives’ knowledge and beliefs on their choices and decisions related to contraception use.

*“Mainly the patient has wrong ideas about contraception. Some of them thought that maybe they will not get pregnant again or that contraception may decrease her chance of pregnancy later.” (Participant #2)*

*“Regarding social attitudes, for example, toward IUDs, the woman may believe that her husband will not be satisfied with her if she uses an IUD, or if she had a cesarean section, then she shouldn’t put in an IUD. So it is just a social effect; she believes social advice more than medical advice.” (Participant #10)*

#### Theme: self-prescribing

Some participants raised the issue of self-prescribing, as some patients visit the pharmacy and choose any contraception without knowing what is the best method and suspected side effects for them.

*“Many studies show that most major side effects are due to self-prescribing because they did not screen for contraindications.” (Participant #1)*

*“Most of the patients start contraception for themselves by going to the pharmacy and buying it, and if they are happy with it, then they probably continue, and the problem is when they get advice and do not consult anybody.” (Participant #5)*

#### Theme: health system organization

Some participants highlighted the issue of a lack of connection between different hospitals and clinics in the country. The patient can visit many physicians and obtain different opinions.

*“Patients go and receive a lot of opinions, and they become confused about their contraception, so patients come to you the next week after they go to somebody else. They say something completely different, then they stop what you gave them. Patients see many physicians, and they receive contradictory opinions. There is no clear path for a patient who visits one physician. Everything works with this physician. No, they just go to different public hospitals, so these are the main difficulties.” (Participant #7)*

#### Theme: physicians’ knowledge and practices

Some participants perceived that some general practitioners (GPs) and family medicine physicians lack knowledge on the different kinds of contraception available and on counseling patients regarding contraception.

*“I think in my perception, family medicine practitioners should know more about counseling because there are a lot of referrals to Ob/Gyns. It is very easy to perform counseling for contraception as family medicine practitioners because not all the patients have the capability to go to a tertiary hospital to see an Ob/Gyn. Contraception can be prescribed by family medicine practitioners if he or she knows how to conduct low-risk pregnancy follow-up and contraception counseling.” (Participant #4)*

*“I’m not satisfied because it’s not organized prescribing; every doctor, when induced to prescribe things, prescribes for all of his patients. He doesn’t tailor contraception for the patient.” (Participant #9)*

### Difficulties hindering protocol development and implementation

Despite the perception of need that participants had, they highlighted a number of factors that make developing and implementing such a protocol difficult. These factors included the availability of contraception, patient factors, the need for research, the need for physician education, and increased load on the hospital.

#### Theme: availability of contraception

Some participants highlighted the issue of contraception availability in the hospital, as not all forms are available in the hospital, such as Implanon and IUDs.

*“The problem is in the availability in the hospital inventory. Sometimes, in some hospitals, you can find a patch but not a ring or oral contraceptives but not an IUD.” (Participant #4)*

*“IUDs are not available in our hospital, so we tell the patient to buy it and bring it with her next time, both the copper and hormonal. For implants, the patient herself buys it and inserts it herself; we don’t do it for them, so we explain this issue for them the first time.” (Participant #12)*

#### Theme: patient factors

Some participants perceived that many patient factors make implementing such protocols difficult. These factors include patient medical history and contraindications, family planning, patient experiences, and preferences.

*“It will be difficult because not all the patients follow the same track. For example, there’s a patient contraindicated for oral contraceptives, but you can put her on an IUD; you don’t have another option or contraindication for systemic anesthesia, so you want to give her local anesthesia, or a patient doesn’t have any contraindication, but she refuses X and prefers Y. So there are patient factors: contraindications, availability, and patient preferences, so for these factors, I don’t think there will be a protocol to follow. However, you can implement a protocol on how to counsel. You need to know if she has a ring, patches, what the failure rate and side effects are with each one of them, duration, and if she needs follow-up. Then, the patient will choose, and according to the contraindications, you will determine the method.” (Participant #4)*

*“It’s hard to implement a protocol because patients make decisions about it. You can’t force it on patients. Some patients have favorable methods of contraception because it is not only hormonal or medication. There are nonhormonal forms of contraception, such as condoms, barriers, sterilization, and IUDs, so we have to individualize our contraception prescription based on patient needs, her family planning and social history, so it is difficult to create a protocol. In addition, by implementing protocols, we’re forcing the patient and implementing a protocol that will be very rigid because a lot of these patients are young and healthy, so what fits for somebody might not fit for somebody else, not because of medical reasons sometimes, just preferences.” (Participant #7)*

#### Theme: need for educating physicians

One participant perceived that a lack of knowledge among physicians limits the development of contraception protocols. Moreover, almost all the participants agreed on the need for educating physicians regarding the importance of contraception protocols. Some indicated that such education should target family medicine physicians and junior physicians.

*“Yes, 100 percent, because this will lead to benefits for the patients and the physicians themselves. Everything has changed, and we have different types of contraception that have been implemented. For example, some contraception is not available in KKUH (King Khalid University Hospital) but is available in private hospitals, and some physicians only hear about it in books and articles. So I think we need education and evidence-based approaches.” (Participant #3)*

*“Yes, because there is lack of important knowledge among doctors, and mainly I see that with junior residents.” (Participant # 2)*

#### Theme: need for research

One participant perceived that there is not enough research and data about Saudi Arabia regarding contraception use, which makes developing such a protocol difficult.

*“To come up with protocols and guidelines, we should understand the counter status, and there should be more recent research and data. I’m not aware about anybody here did data collection and started looking for contraception in the kingdom and did surveys for women about their favorable methods, what’s commonly use here, and what are the factors that make women choose between contraception methods.” (Participant #7)*

#### Theme: increase the load in hospitals

Some participants perceived that the load raised from developing such protocols is the main barrier to developing a contraception protocol. This load is either a working load (such as increasing the number of patients visiting the clinic to obtain prescriptions and the number of departments and people needed to develop the protocol) or a financial load. Some participants highlighted the issues of existing patient loads and the shortages of Ob/Gyn specialists in the institute.

*“The difficulties will be among patients. They will need prescriptions for contraception. Maybe this will overload a governmental hospital because the number of patients will increase.” (Participant # 1)*

“*Overload, because we should counsel regarding the contraception and what type she wants, and the counseling may take thirty minutes. If we implement this to all patients, we won’t finish because we have high turnover and high patient loads in our hospital.” (Participant #2)*

*“Different people will contribute to do input to the protocol, so each person will look at this from his view. So this is where problem lies, especially when you have too many people involved. You will have to involve so many departments. There will be a lot of discussion and arguments. Additionally, you will have to involve nonmedical people to give approval, and that will be complicated.” (Participant #5)*

*“Financial issues: the more patients there are, the higher the cost will be.” (Participant #1)*

## Discussion

Knowledge and practices regarding hormonal contraceptives have continued to increase in both developed and developing countries [30]. In Saudi Arabia, studies on contraceptive use are lacking. Most of the studies conducted in SA have focused on attitudes, use, and knowledge regarding oral contraceptives. Therefore, we were interested in exploring the need for a contraception protocol in SA from the perspectives of Ob/Gyn physicians. To the best of the authors’ knowledge, this study is the first to elicit the perspectives of Ob/Gyn physicians on current contraception prescribing practices, the need for contraception protocols, and the difficulties hindering the development and implementation of contraception protocols in SA. Given the increased use of contraception worldwide, this study described the issues in local contraception practice and local needs. This study showed a need for a contraception protocols to guide the prescribing process and patient counseling without restricting prescribing. However, Ob/Gyn physicians perceived that the development and implementation of such protocols are limited by some factors. These factors include the availability of contraception, the need for more research, the need for educating physicians, some patient-related factors, and the expected increase in workload.

The lack of a contraception protocol could be the cause of the inconsistency in contraception practices revealed by this study. The results indicated a variation in pre-prescription procedures that Ob/Gyn physicians followed. Some participants indicated that they perform a pregnancy test to rule out any pregnancy before prescribing, while others just depend on the patient’s history of her period. Additionally, an ultrasound is performed only if the participant comes in for an IUD. Moreover, the results showed an inconsistency in patient counseling and follow-up with the patients, whereas previous studies in the country indicated a high rate of adverse effects experienced among women from contraceptive use, which leads patients to cease using contraception in some cases [6, 7, 21, 22]. These findings highlight the need for improving current contraception practices. This can be achieved by developing new or implementing international tools with periodic assessment to assure good contraception practices. An example is the WHO guidelines on selected practice recommendations for contraception use [28]. The WHO SPRs list information that should be provided about each contraceptive method to help the patient make a choice about the contraceptive method [28]. This information includes relative effectiveness, correct usage, how the method works, common side effects, health risks and benefits, signs and symptoms that necessitate a return to the clinic, return to fertility after discontinuation, and sexually transmitted infection protection [28]. This information can be used to guide the patient counseling process during clinic visits.

Existing evidence relates the respect of human rights to positive health outcomes. The WHO highlights the importance of ensuring human rights in the provision of contraceptive information and services [29]. In fact, Ob/Gyn physicians in this study perceived that the protocol should guide the prescribing and counseling processes without restricting prescribing to avoid affecting the patient’s rights.

Ob/Gyn physicians in this study highlighted the low contraception literacy the community has on the issue of self-prescribing and the social effects on patient choices and decisions related to contraception use. Previous studies in the country also reported low contraception literacy in Saudi communities and the need for improving community knowledge of contraception [5–10, 23–26]. Additionally, the issue of self-prescribing was previously reported in a study performed by Al Turkki, in which a majority of Saudi women used contraceptive methods without medical advice [26]. Moreover, some studies recommended the initiation of family planning services in the county to improve Saudi community awareness of contraception and to maintain safety [10, 24]. However, self-prescribing issues could be resolved and community literacy about contraception could be improved by implementing new legislation and emphasizing the role of community pharmacists in contraception practice around the country. Some countries, such as the USA and Canada, provide access to hormonal contraception without a prescription. These countries have legislation emphasizing the role of community pharmacists, with the implementation of pharmacists’ prescribing protocols and self-screening questionnaires to ensure the safe use of contraception by the community [31–33]. England also recently initiated a pharmacy contraceptive service through community pharmacies. This service has a four-tiered approach, including ongoing monitoring and supply of repeat OC prescriptions, initiation of OCs via a patient group direction (PGD), ongoing monitoring and management of repeat long-acting reversible contraception (LARC), excluding IUDs, and initiation of LARCs [34].

Another issue that Ob/Gyn physicians reported was contraception knowledge and practices by family medicine (FM) and general practitioners (GPs). They perceived that some GPs and FM physicians lack knowledge of different kinds of contraception available and of counseling patients regarding contraception. This highlights the importance of education and national implementation of WHO guidelines on MEC and SPRs for contraceptive use. In addition, Ob/Gyn physicians in this study were concerned about the lack of connecting patients’ medical records between different hospitals and clinics around the country, which indeed affects patient safety. In fact, on 10.^th^ February 2015, the Saudi National Health Information Center (SNHIC) initiated shared eHealth files, which aim to link hospitals around the country with a system to improve public health and reduce costs (the Shared eHealth File). However, this system does not currently include private hospitals and private clinics [35].

Lack of availability of contraception in the hospital, some patient factors (including medical history, contraindications, family planning, patient experiences, and preferences), the need for research, the need for physicians’ education about the importance of a contraception protocol, and the perception of an increased workload from implementing contraception protocols in the hospital were reported by Ob/Gyn physicians as factors hindering the development and implementation of a contraception protocol in the country. However, the WHO developed a guide for the integration of MEC and SPRs into national family planning guidelines. This guide provides steps for implementation and adaptation based on local needs [29].

This study’s limitation was that the results cannot be externally generalized, as only participants from one tertiary teaching hospital in Riyadh were included in this study. However, the generalizability of data from this study was not intended; rather, researchers sought to ascertain the need for a contraception protocol in the country from the perspectives of Ob/Gyn physicians. In addition, this tertiary teaching hospital provides health services for a large part of the community, which can represent the Saudi community.

## Conclusion

Ob/Gyn physicians reported the need for a contraception protocol that guides the prescribing process and counseling without restricting prescribing. This research described the current practices of contraception in the country and highlighted the factors affecting the development and implementation of such a protocol. The results of this study can be used to facilitate the adaptation and implementation of WHO contraception guidelines on a national scale.

## Data Availability

The datasets supporting the conclusion of this article are available from the corresponding author (Dr. Noha Al Aloola) upon reasonable request.

## Abbreviations

Ob/Gyns: Obstetricians and gynecologists
IUDs: Intrauterine devices
SA: Saudi Arabia
OCs: Oral contraceptives
WHO: World Health Organization
MEC: Medical eligibility criteria
SPRs: Selected practice recommendations
FM: Family medicine
GP: General practitioner
SNHIC: Saudi National Health Information Center

## References

[1] Shrader SP, Ragucci KR, DiPiro JT, Talbert RL, Yee GC, Matzke GR, Wells BG, Posey L (2018). Contraception. Pharmacotherapy: A Pathophysiologic Approach.

[2] Mustafa R, Afreen U, Hashmi HA (2008). Contraceptive knowledge, attitude and practice among rural women. J Coll Physicians Surg Pak.

[3] Rahayu R, Utomo I, McDonald P (2009). Contraceptive use pattern among married women in Indonesia. Kampala, Uganda: International Conference on Family Planning: Research and Best Practices.

[4] Albezrah N (2015). Use of modern family planning methods among Saudi women in Taif, KSA. Int J Reprod Contracept Obstet Gynecol.

[5] Alharbi M, Alharbi M, Alnazzawi A, Albasri R, Al Towairqi M, Alamri W (2016). Knowledge, attitudes and practices towards family planning among Saudi female teachers in Al-Madinah Al-Munawarah City, Saudi Arabia. Int J Acad Sci Res.

[6] Al-Mass AA (2018). User experience, knowledge and practice of oral contraceptive: a study from Riyadh, Saudi Arabia. Ann Med Health Sci Res.

[7] Alhusain F, Alkaabba F, Alhassan N, Alotaibi S, Breakeit S, Musaudi E (2018). Patterns and knowledge of contraceptive methods use among women living in Jeddah. Saudi Arabia Saudi J Health Sci.

[8] Abdel-Salam DM, Albahlol IA, Almusayyab RB, Alruwaili NF, Aljared MY, Alruwaili MS, Alnasser RM (2020). Prevalence, correlates, and barriers of contraceptive use among women attending primary health centers in aljouf region, Saudi Arabia. Int J Environ Res Public Health.

[9] Yasmeen A, Syed MH, Meraya AM, Albarraq AA, Makeen HA, Alqahtani SS, Abubaker M, Syed NKA (2020). Utilization pattern and side effect profile of oral anticonceptives: A community-based cross-sectional study among Saudi women. Int J Clin Pharm.

[10] Mahboub S, Abdelkader S, Al-Muhanna A, Al-Musallam F, Al-Ghannam J, Al-Munyif S (2014). Attitude towards Contraceptives Use among Saudi Women. Int J Health Sci.

[11] Kingdom of Saudi Arabia, General Authority for Statistics. Household Survey 2018. Available online: https://www.stats.gov.sa/en/news/326 (Accessed on 16 Jan 2023).

[12] Avotri JY, Walters V. “You just look at our work and see if you have any freedom on earth”: Ghanaian women’s accounts of their work and health. Soc Sci Med.1999;48 (9):1123–33. 10.1016/s0277-9536(98)00422-5. 10.1016/s0277-9536(98)00422-5 10220014

[13] Strauss JS, Krowchuk DP, Leyden JJ, Lucky AW, Shalita AR, Siegfried EC (2007). Guidelines of Care for Acne Vulgaris Management. J Am Acad Dermatol.

[14] ESHRE Capri Workshop Group (2005). Non Contraceptive health benefits of combined oral contraception. Hum Reprod Update.

[15] Archer DF (2006). Menstrual-cycle-related symptoms: A review of the rationale for continuous use of oral contraceptives. Contraception.

[16] Zahradnik HP, Hanjalic-Beck A, Groth K (2010). Nonsteroidal anti-inflammatory drugs and hormonal contraceptives for pain relief from dysmenorrhea: A review. Contraception.

[17] Robinson SA, Dowell M, Pedulla D, McCauley L (2004). Do the emotional side-effects of hormonal contraceptives come from pharmacologic or psychological mechanisms?. Med Hypotheses.

[18] Gregory S, Hall K, Quast T, Gatto A, Bleck J, Storch E (2018). Hormonal Contraception, depression, and Academic Performance among females attending college in the United States. Psychiatry Res.

[19] Mohamed A, Kelchtermans H, Konings J, van Daal J, Al Marzouki A, Harakeh S (2018). The effects of oral contraceptive usage on thrombin generation and activated protein C resistance in Saudi women, with a possible impact of the body mass index. PLoS One.

[20] White ND (2018). Hormonal contraception and breast cancer risk. Am J Lifestyle Med.

[21] Alsolami FJ, Azzeh FS, Ghafouri KJ, Ghaith MM, Almaimani RA, Almasmoum HA, et al. Determinants of breast cancer in Saudi women from Makkah region: a case-control study (breast cancer risk factors among Saudi women). BMC Public Health. 2019;19(1). 10.1186/s12889-019-7942-3 10.1186/s12889-019-7942-3 PMC6873398 31752790

[22] AlSheef M, Alotaibi M, Zaidi ARZ, Alshamrani A, Alhamidi A, Zaidi SZA, Alanazi N, Alhathlool S, Alarfaj O, AlHazzaa M (2020). Prevalence of cerebral venous thrombosis with the use of oral contraceptive pills during the Holy month of Ramadan. Saudi Med J.

[23] Alsaleem M, Al-musa H, Alfaifi W, Alshumrani Z, Alzuheri N, Aslouf A (2019). Knowledge, attitude, and practice among Saudi primary health care attendees about family planning in Abha, Kingdom of Saudi Arabia. J Family Med Prim Care.

[24] Alsaleem M, Khalil S, Siddiqui A, Alzahrani M, Alsaleem S (2018). Contraceptive use as limiters and spacers among women of reproductive age in southwestern. Saudi Arabia. Saudi Med J.

[25] Al SM (2010). Awareness and use of contraceptives among Saudi women attending primary care centers in Al-qassim Saudi Arabia. Int J Health Sci (Qassim).

[26] Al-Turki H (2011). Contraception: attitudes and experiences of Saudi Arabian women. Health Care Women Int.

[27] WHO (2015). Medical eligibility criteria for contraceptive use.

[28] WHO (2016). Selected practice recommendatios for contraceptive use.

[29] WHO (2018). Implementation Guide for the Medical Eligibility Criteria for Contraceptive Use and Selected Practice Recommendations for Contraceptive Use Guidelines.

[30] WHO. Family planning/contraception methods. Switzerland: WHO. Available from: https://www.who.int/news-room/fact-sheets/detail/family-planning-contraception. [Cited 2020 Nov 9].

[31] Minnesota board of pharmacy. Pharmacist Prescribing Protocols Self-administered Hormonal Contraceptives. Minnesota: Minnesota board of pharmacy. 2020.7p. Available from: https://mn.gov/boards/pharmacy/resourcesfaqs/prescribingprotocols.jsp. [Cited 2020 Dec 24].

[32] Board of pharmacy. Self-Administered Hormonal Contraception Protocol for Pharmacists. California: Board of pharmacy.5p. Available from: https://www.pharmacy.ca.gov/licensees/hormonal_contraception.shtml

[33] Soon JA, Whelan AM, Yuksel N, Rafie S (2021). Enhancing access to contraception through pharmacist prescribing across Canada. Can Pharm J (Ott).

[34] National pharmacy services. Community pharmacy England. Pharmacy Contraception Service.London :Community pharmacy England. 2022. Available from: https://cpe.org.uk/national-pharmacy-services/advanced-services/pharmacy-contraception-service/. [Updated 2023 May 12; Published 2022 September 22].

[35] The Shared E-Health File. Nhic.gov.sa; 2021 Available from: https://nhic.gov.sa/en/Initiatives/Pages/SharedHealthFile.aspx. [Accessed 1 Jul 2021].

